# Supplementary material for: Heads up–Four Giraffa species have distinct cranial morphology
Source: PLoS One. 2024 Dec 19;19(12):e0315043. doi: 10.1371/journal.pone.0315043 (PMC11658530; doi:10.1371/journal.pone.0315043)
Supplement: S1 Appendix — (DOCX) [file pone.0315043.s001.docx]

**S1 Appendix. Institutional abbreviations.**

*Institutional Abbreviations*

Museums: **AMNH** – American Museum of Natural History, New York (USA); **AMPG** – Athens Museum of Palaeontology and Geology (Greece); **DNMNH** – Ditsong National Museum of Natural History, Pretoria (South Africa); **FMNH** – Field Museum of Natural History, Chicago (USA); **HNHM** – Hungarian Natural History Museum, Budapest (Hungary); **ZMB** – Museum für Naturkunde, Berlin (Germany); **NCB** – Naturalis Biodiversity Center, Leiden (the Netherlands); **NHMUK** – Natural History Museum of the United Kingdom, London (United Kingdom); **NHMZ** – Natural History Museum of Zimbabwe, Bulawayo (Zimbabwe); **NMB** – Naturhistorisches Museum Basel (Switzerland); **NMK** – National Museums of Kenya, Nairobi (Kenya); **NMW** – Naturhistorisches Museum Wien (Austria); **RMCA** – Royal Museum of Central Africa, Tervuren (Belgium); **SAM** – Iziko South African Museum, Cape Town (South Africa); **SMF** – Senckenberg Museum Frankfurt (Germany); **SMNS** – Staatliches Museum für Naturkunde Stuttgart, Stuttgart (Germany); **SNM** – Slovak National Museum, Bratislava (Slovakia); **UCT** – University of Cape Town (South Africa); **USNM** – United States National Museum, Smithsonian Institute, Washington DC (USA); **ZFMK** – Zoological Research Museum Alexander Koenig, Bonn (Germany); **ZMUA** – Zoological Museum, University of Athens (Greece); **ZSM** – Zoologische Staatssammlung, Staatlichen Naturwissenschaftlichen Sammlungen Bayerns, Munich (Germany).

African Parks/Reserves: **CHA-ZAK** – Zakouma NP (Chad); **KEN-AMB** – Amboseli NP (Kenya); **KEN-MAM** – Maasai Mara NP (Kenya); **KEN-MER** – Meru NP (Kenya); **KEN-MGE** – Mgeno Conservancy (Kenya); **KEN-RUM** – Ruma NP (Kenya); **KEN-TSE** – Tsavo East NP (Kenya); **KEN-TSW** – Tsavo West NP (Kenya); **NAM-ETH** – Etosha Heights Private Reserve (Namibia); **NER-KOU** – Kouré Giraffe Zone (Niger); **UGA-MUR** – Murchison Falls (Uganda); **ZAM-LUA** – Luangwa Valley (Zambia).

Taxidermy Collections: **NAM-ING** – Ingwe (Namibia), **NAM-NYA** – Nyati (Namibia), **NAM-TAU** – Tau (Namibia); **SA-SI** – Splitting Image (South Africa); **ZIM-TCI** – Trophy Consultants International (Zimbabwe).
