# Supplementary material for: Heads up–Four Giraffa species have distinct cranial morphology
Source: PLoS One. 2024 Dec 19;19(12):e0315043. doi: 10.1371/journal.pone.0315043 (PMC11658530; doi:10.1371/journal.pone.0315043)
Supplement: Attachment — Submitted filename: Rebuttal letter.docx [file pone.0315043.s007.docx]

Dear Editor and Reviewers,

We are pleased to resubmit the reviewed version of our manuscript after making the requested changes recommended by the reviewers. Please find below the list of the changes made and noted accordingly with the line number (L) of the initially submitted manuscript and the reviewers’ comments marked red:

Editor

*Please ensure that your manuscript meets PLOS ONE's style requirements, including those for file naming.*

As far as we can see, our manuscript meets the journal’s requirements. If there is something in particular that needs to be changed, please let us know.

*In your manuscript, please provide additional information regarding the specimens used in your study. Ensure that you have reported human remain specimen numbers and complete repository information, including museum name and geographic location. If permits were required, please ensure that you have provided details for all permits that were obtained, including the full name of the issuing authority, and add the following statement: 'All necessary permits were obtained for the described study, which complied with all relevant regulations.' If no permits were required, please include the following statement: 'No permits were required for the described study, which complied with all relevant regulations.'*

L90 – The statement ‘No permits were required for the described study, which complied with all relevant regulations’ was added.

*We note that you have indicated that there are restrictions to data sharing for this study. PLOS only allows data to be available upon request if there are legal or ethical restrictions on sharing data publicly. For more information on unacceptable data access restrictions, please see http://journals.plos.org/plosone/s/data-availability#loc-unacceptable-data-access-restrictions. Before we proceed with your manuscript, please address the following prompts: a) If there are ethical or legal restrictions on sharing a de-identified data set, please explain them in detail (e.g., data contain potentially identifying or sensitive patient information, data are owned by a third-party organization, etc.) and who has imposed them (e.g., a Research Ethics Committee or Institutional Review Board, etc.). Please also provide contact information for a data access committee, ethics committee, or other institutional body to which data requests may be sent. b) If there are no restrictions, please upload the minimal anonymized data set necessary to replicate your study findings to a stable, public repository and provide us with the relevant URLs, DOIs, or accession numbers. For a list of recommended repositories, please see https://journals.plos.org/plosone/s/recommended-repositories. You also have the option of uploading the data as Supporting Information files, but we would recommend depositing data directly to a data repository if possible.*

L150 – The used code was uploaded on <https://github.com/nkargopoulos/Giraffe-Cranial-3D-GM.git>.

Reviewer 1

*44 Features of display – explain what this means*

L44 – The word ‘(ossicones)’ was added.

*56 - 57 the ossicones of Palaeomeryx (Sansan and Aternay) – Libytherium Sivatherium Giraffokeryx Honanotherium resemble those of Giraffa. – the okapi ossicone is different from Giraffa.*

*Putting Samotherium in its place. Cambridge Scholars Press. Book.*

*The giraffe: parts unknown for Cambridge Scholars Press - 2023. Book With 60 plates. Nikos Solounias.*

L58 – The phrase ‘which is markedly different than the giraffe in terms of ossicone morphology’ was added. Concerning the fossil genera suggested, even though the evolutionary development of ossicones is of interest, in this sentence we are strictly discussing extant forms. For this reason, we could not find relevance in citing the suggested literature source concerning *Samotherium*. However, the citation of Solounias (2023) concerning giraffe anatomy was added.

*74 replace intrasexual with sexual dimorphism*

L74 – The phrase ‘a clear intrasexual difference’ was changed to ‘clear sexual dimorphism’.

*93 the entire study depends on this assumption*

L93 – It is true that this study is based on this statement, but the current ranges of the distinct giraffe subspecies enable us to confidently identify each species based on provenance, since the range overlaps are practically absent. In cases of uncertain identification, specimens were not identified to either subspecies or species-level in order to avoid misidentifications. Therefore, we strongly believe that the taxonomic framework of our dataset is reliable.

*Victoria Lee, Nikos Solounias 2023. Osteological foot differences between giraffes from distinct geographical locations; a pilot study. Zoologishcer Anzeiger* [*https://doi.org/10.1016/j.jcz.2022.01.001*](https://doi.org/10.1016/j.jcz.2022.01.001) *- We got the cover too.*

L275 – The phrase ‘and postcranial’ as well as the citation of Lee and Solounias (2022) were added.

Reviewer 2

*This leads into the abstract in lines 33-35, where I think you could provide a more robust reasoning as to why it is important to understand their evolution, ontogeny and taxonomy. Later in the abstract you tend to focus on conservation and mention taxonomy in the opening lines of the main text, so I would use this as the motivation to then set up the rest of the abstract.*

L35 – The importance of conservation implications was added.

*In the introduction, I do not think that line 55-56 is needed, I think it should be clear that the study is important without saying it explicitly. Instead use this sentence to say why your study offers for an increased understanding of distinguishing between taxa.*

L55 – This sentence was modified to better reflect the significance of the study.

*Lines 73-76 is a little confusing, you say our results, does this refer to this study or another? If a previous one, please cite the study and make this clearer, if this one then I do not think it should be discussed here.*

L73 – The phrase ‘Our results’ was replaced by ‘The results of the latter study’.

*In the methods, I do not think you need table 1 in the main text, I would move this to the supplementary, the shape figures (Fig.1) show the distributions in a round about way. I think that it is great that you included this, but it fulfils a similar role to table S1, and it is not integral to understanding the paper.*

L95 – Table 1 was moved to the Supplementary (now S1 Table).

*In lines 88-89, you mention they were scanned, but then provide more details in the next section (methods). I would integrate materials and methods. A way to circumvent the issue is do not mention scans in line 88, instead say collected.*

L88 – The word ‘scanned’ was changed to ‘sourced.

*The abbreviations in the text in lines 98-124 makes it difficult to navigate the text.*

L98–124 – We appreciate the comment about abbreviations making it difficult to navigate the text. However, since it is mandatory to mention the collections in which each specimen is deposited, we have to add them to the Material and Methods section, and this is a relatively standard way to list the used abbreviations. If the reviewer or the editor want to suggest a different way to present the institutional abbreviations, we will be happy to adjust.

*You mention you use two segments (lines 141-142), so I would give a different colour scheme for each of these modules.*

L141 – The two segments of the landmark protocol were removed from the Material and Methods section.

*You provide details of the landmark scheme in Table S2 which is good, but I suggest that you provide a figure that shows the landmark scheme. I would replace Table 1 with this figure. You could for instance provide a landmark scheme on a single specimen, where you show the landmarks and semilandmarks on the underlying mesh.*

L146 – A figure was added depicting the landmark protocol.

*It would also be great if you uploaded your code and data to a GitHub repo to help reproduce the results in the future.*

L150 – The used code was uploaded on <https://github.com/nkargopoulos/Giraffe-Cranial-3D-GM.git>.

*The next part of the methods is related to ontogeny. I like the idea, but I would question if you had enough repeated specimens across all of the different taxa to make a meaningful insight. You give the age classes in Table S3, but you do not provide the actual number of specimens for each (sub)species that you use, so this makes inference about the meaningfulness of these results difficult. I have no issue with the use of the dental wear, rather my concerns are about sample size.*

L195 – Concerning the number of specimens per taxon for the allometry, we used nearly all the available specimens, except for those that had major damage and we could not accurately predict their centroid size. Therefore, the numbers per taxon are almost identical to those in S1 Table (former Table 1).

*In lines 195-203, you then discuss allometry and relate this to ontogeny. You mention juveniles, but I cannot identify them in the plot.*

L199 – The juveniles are represented on the left side of the regression plots since they have the smallest centroid sizes. We added a note in the text in case this was not clear. However, we would like to retain the juveniles in the plots to present the developmental component of these regressions. Even though adult allometry is interesting, our main point in this part of the manuscript highlights the different ontogenetic trajectories between the north and south clades in males and females, which have to do with the transition from juveniles to adults. Therefore, we propose to keep them in our plots.

*I think it may be best to just focus on the allometry side of things using adults only to understand the results better. I think the statements in lines 202-203 related to geography are hard to see, I think this needs some rethinking of how to show this. The differences between sexes are clearer. Could you do plots instead related to (1) sex and (2) species?*

L202 – Concerning the geographical differences, in Fig. 4B (former Fig. 2B) the green points (north clade) are plotted higher than the blue points (south clade). This was further investigated in Figs. 4C and 4D (former Figs. 2C and 2D), in which the trendlines show the same pattern. Concerning making the regression for species, there aren’t any clear differences between the species of the same clades (reticulated-northern and southern-Masai) and the plots become crowded. That is why we preferred keeping the simpler north-south distinction that shows the clearest differences. Concerning sex, we have regressed males and females showing sex-related allometry (Fig. 4A, former Fig. 2A) and also investigated each sex independently showing sex-specific taxonomy-related allometry (Figs. 4C and 2D, former Figs. 4C and 2D). Please elaborate if the reviewer feels an extra plot is needed.

*Then I would just display the extreme morphologies using one view, rather than 5. This will help keep it easier to follow. Providing the landmarks on the craniums is also useful here to back up statements in lines 187. Could heatmaps using thin plate splines be used to further demonstrate these statements?*

Concerning the visualization of the extreme morphologies in the PCAs and CVAs, even though we realize that the 3D model views occupy a lot of space in the figures, we believe all of them are necessary to depict the changes across the axes: the ossicones (lateral view), the orbits (mesial view), the nuchal crest (distal view), and the rostrum (dorsal view) and are morphed using the Thin Plate Spline. Therefore, we prefer to keep them instead of heatmaps, as they are more expressive of morphology, and therefore easier to be interpreted.

*Could perhaps give an example of each species (mesh or landmarks) next to the convex hull for each species.*

Concerning the addition of mesh examples for each taxon on the plots, given the high number of taxa (8 for the subspecies) the plot becomes quite crowded, and the skulls are too small to see any actual differences. Therefore, we abandoned this option even though it could give a more realistic view of the differences between the taxa. However, some examples are now seen in the new Fig. 1, as well as in the new Fig. 7.

*Table 1 reaffirms the results of these, but it is quite messy and large so may be better suited for the supplementary as Figure 4 shows a lot of these differences. Maybe keeping it just for species (rather than subspecies too) may a way of keeping Table 1 in the main text without it being too hard to read.*

L254 – Table 2 was moved to the Supplementary (now S5 Table).

*Differences in the shape between species are clear in the morphospace plots, and you mention this is mainly due to the ossicones, I think you need a figure somewhere that really demonstrates this (visualising the landmarks will help).*

L273 – An additional figure (Fig. 7) was added showing the differences in lateral view between the four species in males and females, and further described in the paragraph above.

*Also given this, have you thought about looking at the phenotypic integration and modularity in the skulls of these two regions?*

*For the results, I agree with the findings that there are differences between sexes and the clades, but I think you need to be careful with one and the other affecting the other. Instead of having two separate plots, instead I recommend making a single plot that includes data related to sex and geography, this way you can remove the bias of sex on the results for geography (if more males in one than the other) and vice versa. Figure 1 is very busy, so I have some suggestions. Condense to two subplots: (1) a PCA with classes showing both species and sex, (2) a PCA with geography and sex.*

*A couple of additional points outside of the manuscript, would be to think about what you can show given the obtained data. You have lots of shape data and a phylogeny, I think some simple evolutionary studies could be done to give the paper a wider reach and interest. For instance, you could look at phylogenetic signal using geomorph, you could also look at morphological disparity and evolutionary rates across the different species to further aid in understanding the evolution (and by extension the taxonomy) of these organisms. The inclusion of two modules also means integration and modularity can be further added to give more insights into the roles of ossicones in determining differences. Just a few things to think about if you wanted to improve the study with what I think would be little extra work given what is presented here.*

Concerning the additional points suggested by the reviewer (phylogenetic signal, morphological disparity, evolutionary rates, integration and modularity, separate CVAs for the different sexes), we acknowledge that our sample offers a wide range of options for future development of the project. All these suggestions are welcome, and we considered them before submitting the manuscript. In fact, we are currently working on several of them to conduct a more thorough analysis of the allometric and evolutionary implications of the giraffe skull shape. However, we chose to keep the present manuscript straightforward and focus on the conservation implications of the suggested taxonomic scheme. We believe that this approach will offer us the chance to present our results in the best possible way and undertake a deeper analyses in future.

Reviewer 3

*Considering that locality was used as a criterion for taxonomic identification and is an important factor in the debate the article aims to address, I believe that a map showing the distribution of the species and subspecies would greatly enhance the study's clarity.*

L96 – A map showing the distribution of species and subspecies as well as representative male skulls in lateral view for each of the subspecies was added as the new Fig. 1. The numbering of the other figures was adjusted accordingly.

*Even though the CT scan images are not the primary focus of the study, I think it would be good practice to share the CT scan parameters.*

L129 – The CT scan parameters were added.

*I believe an illustrative diagram showing the landmarks would be a great complement to Table 2 in the supporting information. Since you used Stratovan Checkpoint, this might be a bit more challenging to illustrate, but I think it's achievable and would add clarity.*

L146 – A figure was added depicting the landmark protocol.

*You should specify which reference phylogeny you used to create the trees in Mesquite.*

L159 – The phrase ‘based on the phylogenetic scheme of Coimbra et al. (2021)^33^’ was added.

*Instead of writing "same as" I suggest writing out the full legend again for clarity, even if it makes the reading a bit repetitive. This apply to the other legends as well.*

L191 – Instead of writing ‘Same as’ in the legends of repeated plots, the full description was readded. The same was changed for all figures.

*Again, you need to indicate which phylogeny you're referring to in this section.*

L222 – The phrase ‘based on the phylogenetic scheme of Coimbra et al. (2021)^33^’ was added.

*The sentence, "The characteristics of the south clade resemble more the plesiomorphic morphology of fossil Giraffa, which is related to the role of ossicones in their social behavior," needs more context. References to a paper on the morphology of fossil giraffes and a better explanation of the role of ossicones in their social behavior would add clarity.*

L273 – Reference concerning extant giraffe social behavior and ossicone morphology of fossil giraffids were added.

*In the discussion, I believe you could more clearly explain how the IUCN treats the taxonomy of giraffes. It is implied that the IUCN recognizes only one species, but the recognition of subspecies is less clear and could benefit from further elaboration.*

L274 – The current taxonomy followed by IUCN was added.

*I also consider it important to dedicate a section before the conclusion to address the limitations of the study (e.g., the issue of giraffe age mentioned in the methodology) and how future studies could overcome these limitations. Since you will be making the CT scan images available, it would also be worth pointing out that other aspects, such as ecomorphology, could be explored in future research.*

L341 – Some limitation and possible further developments of the project were added.

*In your acknowledgments, you cite the Giraffe Conservation Foundation for their financial support. Should this not be placed in the financial disclosure section?*

Concerning the received funding, GCF financially supported the project directly and the funds did not come from a specific project grant or similar. Mentioning GCF in the acknowledgments aims to thank them not only for the funding to undertake the project (postgraduate fellow, equipment, travel) but also additional GCF personnel involved in the project but not authors.

Personal Changes

L361 – The sentence ‘We are thankful to the editorial board of PLoS ONE and to the three reviewers (N. Solounias and two anonymous reviewers) for their help during the peer review and the production process’ was added.

Very minor changes were made in the use of language at some points in the manuscript.

We thank you again for the review and hope the changes made are more than adequate. Please do contact me if you require for any further comments, and look forward to hopefully seeing the manuscript published soon.

Best regards,

Nikolaos Kargopoulos
