# Supplementary material for: Heads up–Four Giraffa species have distinct cranial morphology
Source: PLoS One. 2024 Dec 19;19(12):e0315043. doi: 10.1371/journal.pone.0315043 (PMC11658530; doi:10.1371/journal.pone.0315043)
Supplement: Attachment — Submitted filename: Rebuttal letter.docx [file pone.0315043.s008.docx]

Dear Editor and Reviewers,

We are pleased to resubmit the reviewed version of our manuscript after making the requested changes recommended by the reviewers. Please find below the list of the changes made with the reviewers’ comments marked red:

Reviewer 2

*The institutional abbreviations may be better suited in the supplementary material rather than main text, unless the authors think it is essential to contain in the main text. They have discussed this comment, so I will delegate to the editor in terms of journal fit.*

The institutional abbreviations were removed from the main body of the manuscript and were placed in a separate document as S1 Appendix.

*The authors have kept the majority of the shape figures the same. I suggested to reduce the size of these figures in terms of the morphological representations, the authors have justified why these have not been changed. I do not think the figures are hard to navigate by any means, therefore, I believe the style of these is best left to the authors and editors.*

We appreciate to be given the choice whether to change or retain the style of our figures. We believe the current presentation works well but are open to comments from the editor.

*The authors have made a significant effort to improve their data accessibility with inclusion of more data and code. I suggest adding the data to the GitHub page upon publication, but I think for publication this is vastly improved. I would still like for the shape data (landmark co-ordinates, either Base or GPA transformed) to be made available for reproducing the results and plots before publication.*

We appreciate the suggestion of the reviewer to make our landmark data publicly available. We also believe that the results of all studies should be accessible for further development. However, we do not wish to share the landmark data yet. All the collaborators have spent valuable time and resources to complete this project and, as mentioned in the previous rebuttal letter, the discussed paper is not the only outcome that will be published out of it. A longer paper that dives deeper into the evolutionary, ontogenetic and taxonomic details of giraffe morphology is currently in preparation and will be submitted next month. We are not comfortable to make all data available before this second manuscript has been accepted for publication. Once this second paper has been published, we will make the landmark data available. As a sign of good faith we have already uploaded the data to GitHub (<https://github.com/nkargopoulos/Giraffe-3D-GM---landmarks>) as a private repository that can easily be made public in due course. In the meantime, if the reviewer wishes to personally check whether our statistical analysis is valid, we would be happy to share the landmark file confidentially and for reviewing purposes only.

*I still have some questions in referring to ontogeny, the authors have clarified in their response of how they use the samples, but I still wonder if you can thoroughly look at ontogeny. Given it is something discussed minimally in the text, I would omit its use and stick with a focus on allometry. I have no problem retaining the juveniles in relation to this.*

As mentioned in the previous comment, we are preparing a more detailed manuscript that will include a thorough analysis of the relationships between allometry and ontogeny. However, since the reviewer considers that the present manuscript would benefit from it, some additional notes and observations were included in the part of the manuscript that discusses ontogeny and allometry to provide more detailed analysis of the results. Also, in some cases the influence of allometry was stated more clearly in order to avoid the misconception that all variability seen in this part of the manuscript is only related to ontogeny.

Reviewer 3

*The only new suggestion I would have is on page 6, line 165, and page 9, line 232. I would replace "phylogenetic scheme" with "phylogenetic hypothesis," but this is merely a stylistic and optional choice.*

In both cases, the work “scheme” was removed with the word “hypothesis”.

We thank you again for the review and hope the changes made are more than adequate. Please do contact me if you require any further comments, and we look forward to hopefully seeing the manuscript published soon.

Best regards,

Nikolaos Kargopoulos
